# Indomethacin counteracts the effects of chronic social defeat stress on emotional but not recognition memory in mice

**DOI:** 10.1371/journal.pone.0173182

**Published:** 2017-03-09

**Authors:** Aránzazu Duque, Concepción Vinader-Caerols, Santiago Monleón

**Affiliations:** Department of Psychobiology, University of Valencia, Valencia, Spain; Technion Israel Institute of Technology, ISRAEL

## Abstract

We have previously observed the impairing effects of chronic social defeat stress (CSDS) on emotional memory in mice. Given the relation between stress and inflammatory processes, we sought to study the effectiveness of the anti-inflammatory indomethacin in reversing the detrimental effects of CSDS on emotional memory in mice. The effects of CSDS and indomethacin on recognition memory were also evaluated. Male CD1 mice were randomly divided into four groups: non-stressed + saline (NS+SAL); non-stressed + indomethacin (NS+IND); stressed + saline (S+SAL); and stressed + indomethacin (S+IND). Stressed animals were exposed to a daily 10 min agonistic confrontation (CSDS) for 20 days. All subjects were treated daily with saline or indomethacin (10 mg/kg, i.p.). 24 h after the CSDS period, all the mice were evaluated in a social interaction test to distinguish between those that were resilient or susceptible to social stress. All subjects (n = 10–12 per group) were then evaluated in inhibitory avoidance (IA), novel object recognition (NOR), elevated plus maze and hot plate tests. As in control animals (NS+SAL group), IA learning was observed in the resilient groups, as well as in the susceptible mice treated with indomethacin (S+IND group). Recognition memory was observed in the non-stressed and the resilient mice, but not in the susceptible animals. Also, stressed mice exhibited higher anxiety levels. No significant differences were observed in locomotor activity or analgesia. In conclusion, CSDS induces anxiety in post-pubertal mice and impairs emotional and recognition memory in the susceptible subjects. The effects of CSDS on emotional memory, but not on recognition memory and anxiety, are reversed by indomethacin. Moreover, memory impairment is not secondary to the effects of CSDS on locomotor activity, emotionality or pain sensitivity.

## Introduction

In recent years, there has been a vast increase in research on how stress affects people lives. An accumulating body of evidence shows socially stressed individuals to be less psychologically and physically healthy [1–3]. Given that social stress is a chronic or recurring factor in the lives of virtually all higher animal species [4], research has recently increased the use of social stress models. In fact, the most adequate and strongest effects on the behavior and physiology of animals living in groups may be obtained through manipulations of socially significant factors [5]. In animals, chronic psychosocial stress can cause elevated cortisol, adrenal gland hypertrophy, hippocampal atrophy, and downregulation of glucocorticoid and mineralocorticoid receptors, as well as depression-like behavioral changes [6]. Among the animal models commonly used, the chronic social defeat stress (CSDS) paradigm has been shown to have excellent etiological, predictive, discriminative and face validity [7], as it induces enduring behavioral and neurobiological changes that mimic several symptoms of the human condition [8–10].

Among the diverse effects produced by social stress, memory impairment is an important negative consequence, as previously shown in a number of animal studies [11–15]. In our laboratory, we have previously observed that CSDS (induced in a 3-week period of 10 min daily sessions) prevented memory formation in post-pubertal mice evaluated in the inhibitory avoidance (IA; also called passive avoidance) test, a common procedure used to evaluate emotional memory in animals [16–18]. These effects of CSDS on memory were not secondary to motor or emotional effects of stress [19–21].

On the other hand, the one-trial novel object recognition (NOR) test is a recognition memory task used to evaluate the behavioral effects of different pharmacological and/or environmental interventions in rodents [22–24]. This test is based on the tendency of rodents to spend more time exploring novel objects than familiar ones and to realize when the position of an object has been changed [25]. It has been demonstrated that stressful situations usually impair object recognition [26, 27].

The effects of stress seem to be partly mediated by inflammatory processes [28], and an increase in inflammation has been reported after repeated social stress [29]. Furthermore, chronic exposure to adverse social environments is associated with stress-related increases in the expression of pro-inflammatory genes, which appear to contribute to a higher risk of disease [30]. Therefore, taking into account the connection between stress and inflammatory processes, it is reasonable to think that anti-inflammatory treatments could have a reversing effect on the alterations produced by CSDS. Indomethacin belongs to the family of non-steroidal anti-inflammatory drugs (NSAIDs), which are available worldwide and frequently used. NSAIDs are safely employed in humans in many inflammatory conditions [31, 32]. It has been demonstrated that indomethacin can attenuate the increased release of stress hormones and neurotransmitters [33] via interleukin 1β, a cytokine which mediates many neurological effects related to inflammation in the brain [34].

Individual differences in stress-regulatory circuits can dramatically affect vulnerability to illness, and maladaptive responses have been implicated in environmental stress in the onset and exacerbation of neuropsychiatric diseases [35]. In fact, experiments using CSDS have revealed large inter-individual variability in subsequent behavioral tests [36, 37], with some animals proving to be resilient to stress. In order to assess this behavioral variability, which can blur the real consequences of CSDS, several tests have been designed to assess sociability in rodents [38], based on the preference of animals to explore social rather than non-social stimuli [8, 39, 40]. Therefore, to evaluate the real effects of CSDS, the social preference-avoidance test for mice was employed [7, 8]. This test identifies animals which are resilient or susceptible to social stress, and some molecular and physiological differences have been identified in these two groups [37].

As already explained, the aim of this study was to assess the effects of chronic indomethacin administration on memory impairment produced by CSDS in post-pubertal male CD1 mice. To achieve this goal, animals were evaluated using the IA test for emotional memory and the NOR test for recognition memory. We predicted that indomethacin would reverse the negative effects of CSDS on memory. Considering that motor activity, anxiety and pain threshold can be confounding factors in animals’ performance in the aforementioned tests (IA and NOR), our animals were additionally assessed in an elevated plus maze and a hot plate test, in which we obtained complementary measures of locomotor activity, emotionality and analgesia.

## Materials and methods

### Subjects

Post-pubertal (42 days) male CD1 mice (Charles River, Lyon, France) were used as experimental subjects. The animals arrived at the laboratory weighing 30–39 g and were housed in groups of 4 in translucent plastic cages (height 14.5 cm, width 27 cm, length 27 cm) with roofs of stainless steel bars (Panlab S.L., Barcelona, Spain). Male CD1 retired breeder mice of over 3 months of age (Janvier, France) were housed individually in similar cages in preparation for their use as aggressors. As in previous studies by our group [19–21], we employed CD1 mice (rather than the commonly used C57BL/6J strain) as stressed animals in the CSDS procedure [7] because using the same strain for the defeated and aggressor subjects enhanced the validity of this model. Furthermore, CD1 is an ideal strain for assessing inhibitory avoidance learning (one of the main memory tests used in the present study). In fact, we have conducted numerous experiments in our laboratory using CD1 mice in this paradigm (see [41]).

All the animals were maintained in a temperature-controlled room (21±2°C) under a reversed light-dark cycle (lights off: 07:30h-19:30h, local time) with food and water available *ad libitum*. Group-housed mice were marked for identification by painting their fur with purple coloring. The animals were subjected to a stress treatment and to several behavioral tests during the dark phase of the cycle. Considering the negative physical consequences of social defeat, such as wounding, adequate measures were taken to minimize pain or discomfort caused to the animals (see Chronic Social Defeat Stress section for details). The experimental protocol and use of animals were in strict accordance with the European Parliament and the Council of the European Union’s Directive of 22 September 2010 (2010/63/EU) and the Spanish Real Decreto 53/2013. All animal experiments were also approved by the Ethics Committee of the University of Valencia (ethical permit number A13281856334).

### Drugs

Indomethacin (Sigma–Aldrich Química, S.A., Madrid, Spain) was dissolved in physiological saline (0.9% NaCl) and administered by intraperitoneal injection (i.p.), at the dose 10 mg/kg, in a volume of 0.01ml/g body weight. The control groups received the same volume of physiological saline.

### Procedure

After 10 days of acclimatization to the animal facility, mice were randomly distributed into four groups: NS+SAL (non-stressed + saline), NS+IND (non-stressed + indomethacin), S+SAL (stressed + saline) and S+IND (stressed + indomethacin).

**Fig 1:**
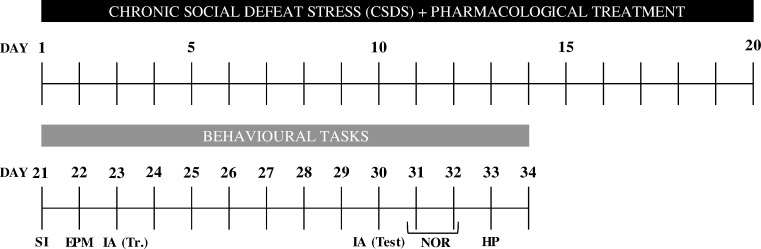
Schedule of CSDS procedure and behavioral tasks performed. SI: social interaction; EPM: elevated plus maze; IA (Tr.): inhibitory avoidance training; IA (Test): inhibitory avoidance test; NOR: novel object recognition; HP: hot plate.

Mice were submitted to a Chronic Social Defeat Stress (CSDS) paradigm, which was used as an animal model of social stress. After the CSDS period, a battery of behavioral tests was applied. A social interaction test was implemented to distinguish between animals that were resilient or susceptible to CSDS (see Social Interaction section for details). All subjects (n = 10–12 per group) were then evaluated in two memory tasks (inhibitory avoidance and novel object recognition tests), as well as in several complementary tasks, in order to control potential confounding variables (elevated plus-maze and hot plate tests). Fig 1 illustrates the time schedule for the experimental design.

#### Chronic social defeat stress

Following a modified version of the guidelines proposed by Golden et al. [7], a Chronic Social Defeat Stress (CSDS) paradigm was used as an animal model of social stress. In this paradigm, experimental male mice (stressed animals) are repeatedly subjected to bouts of social defeat by a larger CD1 mouse that has screened positive for aggressive behavior. In a pre-stress phase, CD1 retired breeders were selected as aggressors based on their attack latencies and the number of attacks they had launched during a 3-day screening procedure (attack latencies shorter than 60 s and 3 or more attacks in 3 min were the aggressor inclusion criteria). In the CSDS phase, the stressed groups were submitted to a daily 10 min social defeat experience by a larger and aggressive mouse on 20 consecutive days. This schedule of CSDS was selected in line with that used in previous studies in our laboratory [19–21] and by other groups (e.g. [11–14]). In the agonistic encounters, each stressed mouse (also called the intruder) was placed in the home cage of an unfamiliar male (the aggressor, also called the resident). All the residents rapidly recognized and launched a first attack against the intruder within 2 minutes. Once the experimental mouse had been physically stressed by defeat during a 10 min period, both animals (intruder and resident) were maintained in sensory contact for 1 h by means of a clear perforated Plexiglas wall that divided the resident’s home cage into two halves. Subsequently, the intruder was returned to its home cage. In each subsequent defeat, experimental mice were exposed to a new resident mouse to counteract any habituation to the resident aggressor and avoid large differences in the level of aggression experienced by the stressed animals. Immediately prior to each agonistic encounter, all stressed animals were injected i.p. with saline or indomethacin (10 mg/kg) according to their experimental condition (S+SAL or S+IND group). Non-stressed mice (NS+SAL and NS+IND groups) were not submitted to any social exposure, but received a daily pharmacological treatment. The body weight of all animals was monitored before and after the CSDS procedure. Every agonistic encounter was continuously supervised by the investigator. Considering that social defeat involves physical aggression, wounding of defeated mice was evaluated daily by veterinary personnel. Following their recommendation, the duration of the defeat sessions was reduced to 5 min (for all animals) from day 17 on. Four intruders had to be removed from the experiment and immediately euthanized in line with the criterion of open wounds exceeding 1 cm, as indicated in the protocol of Golden et al. [7]. Wounds not exceeding 1 cm were observed in forty per cent of the mice that were not removed (these wounded animals represented 36% of the resilient group and 44% of the susceptible group). In order to minimize the discomfort of these animals, their wounds were treated daily with Betadine.

#### Social interaction

Twenty-four hours after the last CSDS session, animals were submitted to a social interaction test for mice, to distinguish between animals that were resilient and those that were susceptible to CSDS [7, 8]. This two-step test took place in an open field arena made of clear Plexiglas (height 35 cm, width 30 cm, length 40 cm). There are two important areas in the open field arena: the ‘interaction zone’, which encompasses a 12 cm × 16 cm rectangular area projecting 8 cm around a circular perforated cylinder, and the ‘corner zones’, which encompass two 9 cm × 9 cm areas projecting from the two corner joints opposite the perforated cylinder [7]. In the first 2.5 min session, the experimental mouse was able to freely explore the neutral area. The perforated cylinder (non-social stimulus) remained empty during that first trial (target absent). The animal was then removed from the open field arena and returned to its home cage for 30 s. During this short inter-exposure interval, an unfamiliar male mouse was placed in the perforated cylinder (social stimulus). In the second 2.5 min trial (target present), the experimental mouse was reintroduced into the arena containing the social target within the perforated cylinder. In this test, the mice used as social stimulus were the CD1 mice used as aggressors in the CSDS procedure, having certified that each target had not been previously paired with the defeated subject throughout the CSDS sessions. The reason for using these animals was to ensure that they emitted similar olfactory, visual, and auditory stimuli as in the CSDS period. The cylinder and the open field were cleaned after each session with a solution of ethanol and water. All sessions were recorded with a video camera (Sony DCR-SR35) for subsequent analysis.

The behavior displayed by the mice during the test was analyzed by a blind researcher using ‘Raton‐time’ software, a program for ethological analysis. A social interaction ratio (SI ratio) is obtained by dividing the time spent in the interaction zone when the target is present by the time spent in that zone when the target is absent. A SI ratio equal to 1, in which equal time is spent in the presence versus the absence of a social target, is used as the threshold for dividing defeated mice into resilient and susceptible categories [8, 37]. Mice scored below this criterion are grouped as susceptible, whereas mice scored above this criterion are grouped as resilient [7]. This means that resilient animals remain longer in the interaction zone when the target is present than when it is absent, which reflects a tendency to explore social stimuli, similar to the behavior seen in non-stressed mice. In contrast, susceptible animals remain in this zone for less time when the target is present than when it is absent, which reflects social avoidance as a result of social stress.

#### Inhibitory avoidance

A one-trial step-through inhibitory avoidance apparatus for mice (Ugo Basile, Comerio-Varese, Italy), contained within an isolation box, was employed to evaluate emotional memory. This cage is made of Perspex sheets and is divided into two compartments (both with a height of 15 cm, width of 9.5 cm, and length of 16.5 cm) separated by a partition with an automatically-operated sliding door. The floor is made of 48 stainless steel bars with a diameter of 0.7 mm and situated 8 mm apart. The safe compartment is white and continuously illuminated by a light fixture fastened to the cage lid (24 V, 10 W, light intensity of 290 lux at floor level, measured with the Panlux Electronic 2 photometer manufactured by GOSSEN, Nürnberg, Germany), whereas the ‘shock’ compartment is made of black Perspex panels and is maintained in darkness at all times.

This task consisted of two phases: training and test. The training phase began with a 90 s adaptation period in the light compartment of the apparatus. Following this, the door between the compartments was opened and the time taken to enter the dark compartment—defined as latency—was automatically measured in tenths of a second and manually recorded. The mouse was allowed to remain in the light compartment for a maximum of 300 s after the door had opened. As soon as the animal entered the dark compartment, the sliding door was closed and a foot-shock (0.3 mA for 5 s) was delivered through the grid floor. The test phase took place one week later, following the same procedure as in the training phase, with the exception that no shock was delivered.

#### Novel object recognition

A novel object recognition (NOR) test was carried out to evaluate the natural preference of the mice for novel objects. An open field arena (height 35 cm, width 30 cm, length 60 cm) made of translucent Plexiglas was employed for this test.

Following the procedure reported by Antunes and Biala [42], the NOR protocol consisted of three phases: (1) Habituation, in which mice were habituated to exploring the open field for 5 min; (2) Training (1 day after Habituation), in which two identical novel objects (two small bottles) were placed in the arena and mice were able to explore them for 10 min; and (3) Test (1 h after Training), in which mice explored a novel object and a familiar one previously explored for 5 min. All objects and the open field cage were cleaned with a solution of ethanol and water after each session. The location of the objects was varied across the treatment groups in order to avoid preference for location. Following the protocol used by other authors (e.g. [25]), all test sessions were recorded with a video camera (Sony DCR-SR35) and the behavior displayed by mice during the test was analyzed by a blind researcher using ‘Raton‐time’ software. An object was considered to have been explored when the head of the animal was 0.5 cm from the object or when it touched the object. In contrast, exploration was considered not to have taken place when an animal climbed onto an object or used it as a base to explore the environment [23]. A discrimination index (total time spent exploring the novel object/total time devoted to exploration of both objects) was calculated for each group to measure recognition memory during the test phase. In addition, the exploration time of the novel and familiar objects within the same group was obtained as another measure of recognition memory [42].

#### Elevated plus maze

All the animals were evaluated in an elevated plus maze for mice (Cibertec, Madrid, Spain), as a complementary behavioral test to measure unconditioned anxiety-like behavior and locomotor activity, following the same protocol as in previous studies in our laboratory (e.g. [19–21, 43]). This apparatus consists of two open arms (30 x 5 cm2 each) and two enclosed arms with walls (30 x 5 x 15 cm3 each) that extend outwards from a common central square (5 x 5 cm2). The maze is made of Plexiglas (black floor and walls) and is elevated to a height of 40 cm above floor level.

This task consisted of a 5 min session that began by placing the mouse in the central square (facing one of the open arms). All sessions were recorded with a video camera (Sony DCR-SR35) for subsequent analysis. The number of entries into the open and closed arms (entry is defined as all four paws being placed on an arm) was scored by a trained observer who was unaware of the treatment applied. Based on former studies [44–46], these scores provide an uncontaminated measurement of locomotor activity through the number of closed arm entries, and one primary anxiety index through the percentage of open arm entries (the lower the score, the higher the anxiety).

#### Hot plate

A hot plate test for mice (Mod Socrel DS37, Ugo Basile, Varese, Italy) was applied to assess nociceptive information. The apparatus consisted of a metal plate (25 x 25 cm^2^) located above a thermoregulator and a plastic cylinder (height 18 cm and diameter 19 cm) made of Plexiglas.

The metal plate was heated through a thermoregulator to a fixed temperature of 55°C (the surface temperature was continuously monitored). Each mouse was placed on the hot plate inside a plastic cylinder to confine it to the heated surface. The latency to lift one or both hind paws was recorded in seconds (s) and provided a nociceptive measurement (the lower the score, the higher the nociception). Animals that failed to lift their paws within 45 s were removed from the plate (to avoid thermal injury) and were assigned a response latency value of 45 s.

### Statistical analyses

As suggested by Golden et al. [7], social interaction data were analyzed by one-way ANOVA for the social interaction ratio and a 2×3 ANOVA was used to compare the interaction zone and corner zones times between target absent and target present in control, resilient and susceptible mice; followed by Tukey post hoc tests. Student’s t tests for dependent samples were performed for comparisons within the same group.

Inhibitory avoidance data were transformed into proportion (p = x/300) values and then to arc sin (arc sin √p) values according to Snedecor and Cochran [47]. This transformation is appropriate when a cut-off time is applied, and crossing latencies that exceed this limit are interpreted as the maximum trial length. Therefore, all latencies are transformed into a percentage or proportion value, and these percentages (p) are then transformed to arc sin (degree) values (according to the formula: arc sin √p) prior to statistical analysis and graphical constructions. Two-way ANOVAs for training and test phases were performed separately. Tukey tests were employed for post-hoc comparisons. Given that inhibitory avoidance learning is defined as statistically significant differences between training and test latencies, a Student’s t test for dependent samples was also carried out in order to check whether each group had learned the inhibitory avoidance task.

Recognition memory in the test phase of the novel object recognition test was measured using a discrimination index (total time spent exploring the new object/total exploration time). One-way ANOVAs were performed to assess differences between groups. Student’s t tests for dependent samples were employed to compare the exploration time of the novel and the familiar object within the same group in the test phase.

After checking that data fulfilled the criteria for normality and homogeneity, one-way ANOVAs were also carried out for the anxiety, locomotor activity and nociception data obtained in the elevated plus-maze and hot plate tests.

The lack of outliers in the sample data was confirmed using the *Outlier calculator* online tool from ‘GraphPad’ software (https://graphpad.com/quickcalcs/Grubbs1.cfm). The rest of analyses were performed using the IBM SPSS software package, version 22 for Windows [48].

## Results

### Social interaction

Table 1 summarizes the results obtained in the social interaction test.

**Table 1:** Effects of CSDS on the behavior of male post-pubertal CD1 mice in the social interaction test.

Behavioral categories	Stress and pharmacological treatments					
**Social Interaction ratio**	**Non-stressed**		**Stressed-resilient**		**Stressed-susceptible**	
	1.7 ± 0.21		1.5 ± 0.10		0.5 ± 0.06^&&&^^###^	
	Saline	Indomethacin	Saline	Indomethacin	Saline	Indomethacin
	1.5 ± 0.27	1.9 ± 0.31	1.6 ± 0.15	1.3 ± 0.09	0.6 ± 0.06	0.5 ± 0.11
**Time in interaction zone**	**Non-stressed**		**Stressed-resilient**		**Stressed-susceptible**	
	40.7 ± 2.65		56.5 ± 3.17^&^		56.4 ± 2.67^&^	
*Target absent*	Saline	Indomethacin	Saline	Indomethacin	Saline	Indomethacin
	42 ± 3.6	39.4 ± 4.05	55.1 ± 5.13	58 ± 3.77	55,8 ± 3.06	57 ± 4.54
*Target present*	**Non-stressed**		**Stressed-resilient**		**Stressed-susceptible**	
	84.9 ± 4.25		79.8 ± 5.34		27.2 ± 2.97^&&&^	
	Saline	Indomethacin	Saline	Indomethacin	Saline	Indomethacin
	82.4 ± 6.42***	87.4 ± 5.8***	84.7 ± 8.48**	74.5 ± 6.31*	31.9 ± 3.56***	22.5 ± 4.44***
**Time in corner zones**	**Non-stressed**		**Stressed-resilient**		**Stressed-susceptible**	
	18.6 ± 1.8		13 ± 1.25^&^		14 ± 1.63	
*Target absent*	Saline	Indomethacin	Saline	Indomethacin	Saline	Indomethacin
	18 ± 1.78	19.3 ± 3.22	13.8 ± 1.82	12.1 ± 1.77	14.8 ± 2.4	13.3 ± 2.3
*Target present*	**Non-stressed**		**Stressed-resilient**		**Stressed-susceptible**	
	6.8 ± 1.07		9.2 ± 2.07		28.6 ± 4.83^&&&^^###^	
	Saline	Indomethacin	Saline	Indomethacin	Saline	Indomethacin
	7.3 ± 1.77**	6.3 ± 1.27*	9.8 ± 3.25	8.6 ± 2.61	30.7 ± 8.58*	26.5 ± 4.89*

**Non-stressed** (n = 20) = Non-stressed-Saline (n = 10) + Non-stressed-Indomethacin (n = 10); **Stressed-resilient** (n = 23) = Stressed-resilient-Saline (n = 12) + Stressed-resilient-Indomethacin (n = 11); **Stressed-susceptible** (n = 20) = Stressed-susceptible-Saline (n = 10) + Stressed-susceptible-Indomethacin (n = 10). Values are expressed as means (±SEM).

&p<0.05

&&&p<0.001 vs Non-stressed group

###p<0.001 vs Stressed-resilient group

*p≤0.05

**p<0.01

***p<0.001 vs target absent.

#### Social interaction ratio (SI ratio)

One-way ANOVA analyses revealed an effect of the factor Stress (*F*_(2,57)_ = 21.727, *p <* 0.001), with susceptible mice obtaining a significantly lower SI ratio value than non-stressed or resilient subjects (*p <* 0.001 in both cases). Neither the factor Drug nor the interaction Stress X Drug was statistically significant (*F*_(1,57)_ = 0.001, n.s.; *F*_(2,57)_ = 1.773, n.s.; respectively).

#### Time spent in the interaction zone

The factor Stress was significant in the time spent in the interaction zone when the target was absent (*F*_(2,57)_ = 9.331, *p <* 0.001), with resilient and susceptible subjects staying significantly longer in this zone than non-stressed subjects (*p <* 0.01 in both cases). Neither the factor Drug nor the interaction Stress X Drug was statistically significant (*F*_(1,57)_ = 0.021, n.s.; *F*_(2,57)_ = 0.224, n.s.; respectively).

The factor Stress was also significant in this measure when the target was present (*F*_(2,57)_ = 50.207, *p <* 0.001), with susceptible subjects spending significantly less time in this zone than non-stressed and resilient subjects (*p <* 0.001 in both cases). Neither the factor Drug nor the interaction Stress X Drug was statistically significant (*F*_(1,57)_ = 0.906, n.s.; *F*_(2,57)_ = 0.896, n.s.; respectively).

#### Time spent in the corner zones

The time spent in the corner zones when the target was absent was dependent on an effect of the factor Stress (*F*_(2,57)_ = 3.585, *p <* 0.05), with resilient subjects staying significantly less time in this zone than non-stressed subjects (*p <* 0.05). Neither the factor Drug nor the interaction Stress X Drug was statistically significant (*F*_(1,57)_ = 0.124, n.s.; *F*_(2,57)_ = 0.258, n.s.; respectively).

The time spent in the corner zones when the target was present was also dependent on an effect of the factor Stress (*F*_(2,57)_ = 14.546, *p <* 0.001), with susceptible subjects spending significantly more time in this zone than non-stressed and resilient subjects (*p <* 0.001, in both cases). Neither the factor Drug nor the interaction Stress X Drug was statistically significant (*F*_(1,57)_ = 0.356, n.s.; *F*_(2,57)_ = 0.081, n.s.; respectively).

#### Interaction zone with absent vs present target

Non-stressed and resilient animals spent more time in the interaction zone when the target was present (NS+SAL: *p <* 0.001, NS+IND: *p <* 0.001, RES+SAL: *p <* 0.01, and RES+IND: *p <* 0.05), whereas susceptible subjects spent less time in said zone when the target was present (SUS+SAL: *p <* 0.001; SUS+IND: *p <* 0.01).

#### Corner zones with absent target vs present target

Non-stressed animals spent less time in the corner zones when the target was present (NS+SAL: *p <* 0.01; NS+IND: *p <* 0.05), whereas susceptible subjects spent more time in these zones when the target was present (SUS+SAL: *p =* 0.05; SUS+IND: *p <* 0.05).

### Inhibitory avoidance

#### Training phase

Neither of the main factors–Stress or Drug–was statistically significant (*F*_(2,57)_ = 0.841, n.s.; *F*_(1,57)_ = 0.004, n.s.; respectively), and nor was their interaction (*F*_(2,57)_ = 0.962, n.s.).

#### Test phase

Neither of the main factors–Stress or Drug–was statistically significant (*F*_(2,57)_ = 1.097, n.s.; *F*_(1,57)_ = 1.13, n.s.; respectively), and nor was their interaction (*F*_(1,57)_ = 1.283, n.s.).

#### Training phase vs test phase

**Fig 2:**
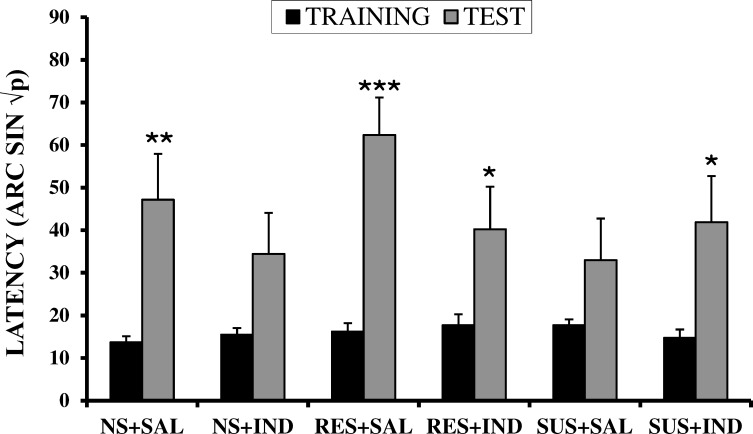
Effects of CSDS and indomethacin on latencies of an inhibitory avoidance task in post-pubertal male CD1 mice. NS+SAL = Non-stressed + Saline group (n = 10); NS+IND = Non-stressed + Indomethacin group (n = 10); RES+ SAL = Resilient + Saline group (n = 12); RES+IND = Resilient + Indomethacin group; (n = 11); SUS+SAL = Stressed + Saline group (n = 10); SUS+IND = Stressed + Indomethacin group (n = 10). Values are expressed as means (+SEM) of square root of proportions (p = x/300) transformed to arc sin. *p<0.05, **p<0.01, ***<0.001 vs Training phase.

Comparing training and test latencies in each group, IA learning was observed in NS+SAL (*p <* 0.01), RES+SAL (*p <* 0.001), RES+IND (*p <* 0.05) and SUS+IND (*p <* 0.05) groups, but not in NS+IND and SUS+SAL (*p >* 0.05 in both cases) groups (Fig 2).

### Novel object recognition

**Fig 3:**
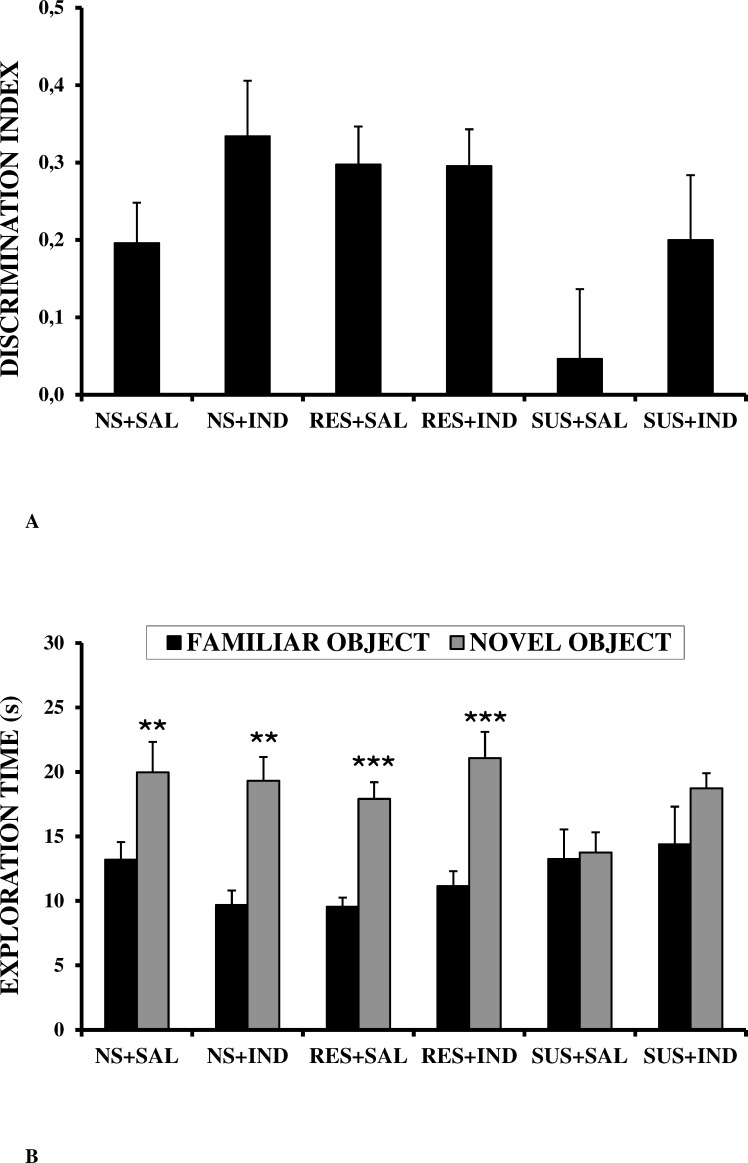
Effects of CSDS and indomethacin on a novel object recognition task by post-pubertal male CD1 mice. A) Discrimination index; B) Exploration time of novel object vs familiar object. NS+SAL = Non-stressed + Saline group (n = 10); NS+IND = Non-stressed + Indomethacin group (n = 10); RES+ SAL = Resilient + Saline group (n = 12); RES+IND = Resilient + Indomethacin group; (n = 11); SUS+SAL = Stressed + Saline group (n = 10); SUS+IND = Stressed + Indomethacin group (n = 10). Values are expressed as means (+SEM). **p<0.01, ***p<0.001 vs familiar object.

In the discrimination index, neither Stress nor Drug was statistically significant (*F*_(2,57)_ = 2.347, n.s.; and *F*_(1,57)_ = 3.758, n.s.; respectively), and nor was the Stress X Drug interaction (*F*_(2,57)_ = 1.102, n.s.) (Fig 3A).

However, the student’s t tests for dependent samples revealed that recognition memory (exploration time of the novel object significantly higher than exploration time of the familiar one) was observed in NS+SAL (*p <* 0.01), NS+IND (*p <* 0.01), RES+SAL (*p <* 0.001) and RES+IND (*p <* 0.001) groups, whilst recognition memory was absent in S+SAL and S+IND (*p >* 0.05 in both cases) groups (Fig 3B).

### Elevated plus maze

#### Locomotor activity (number of entries in enclosed arms)

**Fig 4:**
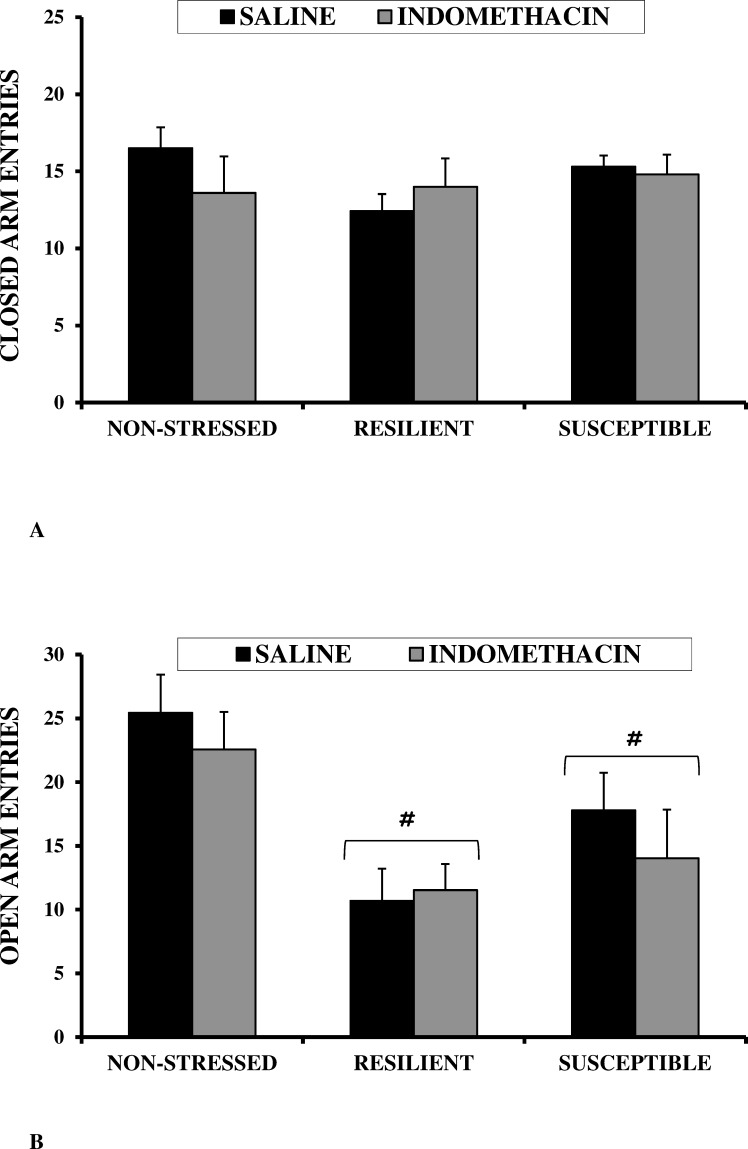
Effects of CSDS and indomethacin on measurements of post-pubertal male CD1 mice in an elevated plus maze task. A) locomotor activity measurement (number of closed arm entries); B) anxiety measurement (percentage of open arm entries). Values are expressed as means (+SEM). #*p*<0.05 vs Non-stressed.

Neither Stress nor Drug was statistically significant (*F*_(2,57)_ = 1.733, n.s.; and *F*_(1,57)_ = 0.403, n.s.; respectively), and nor was the interaction Stress X Drug (*F*_(2,57)_ = 1.882, n.s.) (Fig 4A).

#### Anxiety (percentage of open arm entries)

One-way ANOVA revealed significant differences in anxiety; the main factor Stress proved to be statistically significant (*F*_(2,57)_ = 10.259, *p <* 0.001) and significantly higher levels of anxiety (lower scores) were detected in both resilient (*p <* 0.05) and susceptible (*p <* 0.05) subjects vs. non-stressed subjects (Fig 4B). Neither the factor Drug nor the Stress X Drug interaction was statistically significant (*F*_(1,57)_ = 0.668, n.s.; and *F*_(2,57)_ = 0.37, *ns*; respectively).

### Hot plate

**Fig 5:**
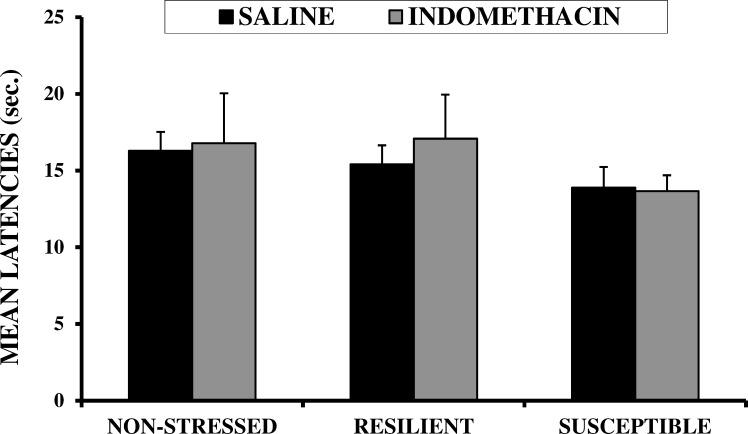
Effects of CSDS and indomethacin on latencies to lift hind paws in post-pubertal male CD1 mice in a hot plate task. Values are expressed as means (+SEM).

No statistically significant differences were observed for either of the main factors, Stress or Drug (*F*_(2,57)_ = 1.083, n.s.; *F*_(1,57)_ = 0.153, n.s.; respectively), or their interaction (*F*_(2,57)_ = 0.116, n.s.) (Fig 5).

## Discussion

The aim of the present study was to evaluate the effects of CSDS on emotional and recognition memory in post-pubertal male mice, using the CD1 strain for the stressed subjects instead of the C57BL/6J strain used in the CSDS standard protocol, and to assess the effectiveness of the anti-inflammatory indomethacin in reversing the said effects. Overall, both types of memory were impaired by CSDS; however, while chronic indomethacin treatment counteracted emotional memory impairment, it did not do so with respect to recognition memory impairment.

Behavioral processes and their underlying physiological systems vary throughout life. Adolescence in both rodents and humans is a period of ongoing refinement and maturation of neural circuitry, which continues into adulthood [49]. Therefore, this developmental stage is a time of vulnerability to stressors that can alter neurobehavioral processes [50]. Late adolescence is a period in which these changes still occur. Considering this and following previous results from our research evaluating the effects of CSDS at different life stages, from which we obtained stronger effects on post-pubertal animals (see [19, 20]), we chose to continue our research with mice from this age group.

It has been established that social stress is a risk factor for affective disorders in vulnerable individuals [51]. This means there can be variability in behavior within a group of laboratory animals exposed to the same stressor, and so a stressor should be combined with a coping or social exploration test [52]. As expected, in this study the social interaction test allowed us to distinguish between animals that were resilient or susceptible to social stress based on the SI ratio (time spent in the interaction zone with target vs. without target), obtaining 53.5% of resilient and 46.5% of susceptible mice. Our data reveal that, with social stimuli (with the target present), resilient and non-stressed mice spent more time in the interaction zone and less time in the corner zones than susceptible animals. Similar to non-stressed mice, resilient animals remained in the interaction zone for a longer period when the target was present than when it was absent; however, susceptible subjects remained in the interaction zone for less time and spent longer periods in the corner zones when there was social stimuli. These data are consistent with previous studies [8, 37, 53] in which the social defeat stress paradigm induced two phenotypic responses during a subsequent social interaction test: resilient mice did not display a decrease in social interaction after social defeat, in a similar way to the non-stressed group, whereas susceptible mice were characterized by prolonged social avoidance, even though all animals had an identical genetic background and all had been exposed to similar conditions of social defeat stress. Therefore, resilient animals displayed a similar behavioral pattern to that of non-stressed mice. It is well established to study the effects of social stress distinguishing resilient versus susceptible subjects based on their behavior in the social interaction test. However, given that wounding is observed in defeated animals, it could be that wounded mice were behaviorally distinct from non-wounded mice and, therefore, resilient/susceptible phenotypes arise from non-wounded/wounded condition. Nevertheless, taking into account that the percentage of wounded animals in the resilient and the susceptible groups were similar (around forty per cent), we think that the social interaction test is a suitable tool for distinguishing between resilient and susceptible individuals.

In our study, similar to the non-stressed animals treated with saline, IA learning was observed in the resilient groups, as well as in the susceptible mice treated with indomethacin. In contrast, non-stressed animals treated with indomethacin and susceptible mice treated with saline did not show IA learning. These results mean that CSDS prevented IA conditioning. Several studies have reported that CSDS impairs performance in some memory and learning tasks in several species, including tree shrews [11, 12], rats [13, 15] and mice [14]. Previous studies carried out in our laboratory using the CSDS paradigm [19–21] have shown that social stress impairs later IA conditioning in post-pubertal mice and can even impede stressed subjects from learning the task, thereby confirming the detrimental effects of CSDS on emotional memory. Considering that the neural circuits of the amygdala and its connected brain areas are thought to be essential for emotional learning [54], this structure could be a principal brain region involved in the effects of CSDS on IA learning. It has been established that some emotional experiences mediated by the activation of the amygdala are better remembered (e.g. [55, 56]); however, there is accumulating evidence indicating that local inhibitory circuits in the amygdala contribute to, or even mediate, important aspects of emotional memory extinction [57, 58]. Therefore, it is also possible that CSDS principally activates these fear-inhibiting pathways, which would have prevented IA learning in the stressed groups in our studies.

Other studies have demonstrated that CSDS leads to increased emotional learning and memory; e.g. in Pavlovian fear conditioning [59, 60]. However, there is also evidence of fear conditioning impairment produced by CSDS [61], as well as other social stress paradigms, such as social instability [62] and social isolation [63]. A lack of effects of CSDS on IA learning has also been reported [61]. This discrepancy could be partly due to differences in the age of subjects when they were submitted to social stress and when their memory performance was tested. Deficits in emotional memory are usually observed when social stress is experienced by young subjects but evaluated in adult years, whereas improvements are usually observed when the animals are stressed in adulthood. A further explanation for the differential results found in the literature could stem from the degree of stress experienced. According to the classic Yerkes-Dodson inverted U-shaped performance law [64], there is an optimal amount of arousal for performance, and when arousal or stress is lower or higher than this optimal level, performance is poorer. Unlike other studies using low degrees of stress, the degree in this study (which was rather high) would be in the descending limb of the inverted U-shaped curve, which should result in memory impairment.

In the NOR test, our results revealed a tendency for a lower discrimination index in the susceptible animals treated with saline, but no significant differences were observed between the groups. In the same way, Mesa-Gresa et al. [65] also failed to find effects of social stress in the NOR task using this measure. On the other hand, the analyses of the total time spent by animals in exploring both objects during the test phase revealed that non-stressed and resilient groups spent longer investigating the novel than the familiar object, whereas susceptible animals spent a similar amount of time exploring both objects. This result reflects that social stress impairs recognition memory in susceptible subjects. It is recognized that the effects of stress are partly mediated by the action of glucocorticoid hormones on the hippocampal neurogenesis [66]. Several explanations of the neurobiology of the effects of stress on NOR have been proposed, such as neuritic atrophy, reduced neurogenesis and decreased neurotrophin levels in the hippocampus, and an increase of glucocorticoids [52, 67]. Actually, this structure plays an important role in object recognition memory [42]. Some studies have linked chronically elevated stress-hormone levels to hippocampal disruption and subsequent cognitive impairment (e.g. [68–70]). Moreover, studies in humans have demonstrated that elevation of cortisol levels, as occurs with chronic stress or illness, induces memory impairment in a dose-dependent manner [68].

Social stressors are the main source of stress in humans and contribute to the development and expression of diverse pathologies, representing a major risk factor for depression (e.g. [71]). Interestingly, a proportion of subjects exposed to chronic social stress does not show any signs of psychopathology and maintains physiological stability, i.e. allostasis [72]. Therefore, to study the differential effects of sustained social stress, it is very important to distinguish resilient and susceptible individuals. In this study, these two different phenotypes were identified by a social interaction test and these groups, displaying different social behavior, also evidenced different patterns in the memory tests. Moreover, the resilient subjects behaved similarly to the non-stressed animals, as if they had not been stressed. It is important to note that, in addition to the behavioral differences between resilient and susceptible subjects, other behavioral or neurobiological measures may vary between phenotypes, which could be potential indicators of vulnerability to stress. Therefore, further research is needed to elucidate the relative contribution of epigenetic, genetic, and environmental factors to the emergence of these phenotypes.

Glucocorticoid stress hormones are crucially involved in modulating mnemonic processing of emotionally arousing experiences [73, 74]. Furthermore, glucocorticoids are crucial regulators of inflammation and immunity [28] and a growing body of literature suggests that stress significantly impacts on many facets of neuroimmune function [75]. So, it is to be expected that stress has pro-inflammatory effects [76] that may be counteracted by anti-inflammatory treatments. The nonsteroidal anti-inflammatory drug indomethacin has been found to effectively reduce levels of pathophysiologically increased inflammation [77] and to exert a positive impact on neurogenesis in pathophysiological inflammation-associated models [77, 78]. Therefore, considering the relationship between glucocorticoids, inflammation and memory, we expected to observe a favorable effect of indomethacin on the detrimental consequences of CSDS on memory in mice, partly mediated by inflammatory processes. Among the stressed susceptible animals, those receiving indomethacin, unlike those that received saline, learned the IA task (emotional memory) to the same extent as the non-stressed mice receiving saline. This shows that chronic indomethacin treatment was effective in attenuating the emotional memory impairment produced by social stress in susceptible subjects. Given that wounding occurs with CSDS, it is difficult to establish if the behavioral effects of indomethacin are due to its peripheral or central actions, based only on evidence from our study in which we have exclusively studied behavioral measures. Nevertheless, studies that have not involved CSDS (and therefore without wounding) and in which indomethacin has been intraperitoneally administered have reported behavioral effects of this drug on the inhibitory avoidance task (e.g. [79]) and the Morris water maze (e.g. [80, 81]). Other studies without CSDS but using intrahippocampal administration of indomethacin also found this drug to have behavioral effects (e.g. [82, 83]). Furthermore, it has been reported that social defeat activates the immune system, producing lasting changes in peripheral and central inflammation parameters [84]. Indeed, Fuertig et al. [60], who used a modified protocol of CSDS with minimal physical wounding, observed higher blood levels of inflammation parameters in the amygdala and the hippocampus of stressed animals Taking into consideration the aforementioned studies, we believe the effects of indomethacin on memory observed in our study were mediated more by its central actions than its peripheral actions.

In contrast to emotional memory, this treatment was not effective in counteracting the NOR task impairment produced by CSDS in relation to recognition memory. These results could be explained by the specific brain areas implicated in the NOR task, among which, in addition to the hippocampus, the perirhinal cortex, a cortical area adjacent to the hippocampus, also plays a major role. These structures are highly integrated, but while the perirhinal cortex is involved in object recognition after short retention intervals, the hippocampus is responsible for long-term object recognition [85]. Therefore, a longer retention interval between training and test phases is probably necessary to observe the reversing effects of indomethacin on the NOR task impairment observed in our study. Indeed, there are inconsistencies in the literature regarding the effectiveness of indomethacin in reversing inflammation in the hippocampus. Although it has been shown to be effective in enhancing learning and memory by facilitating hippocampal synaptic transmission (see [86]), some studies have found that indomethacin is unable to effectively attenuate neuroinflammation and protect neurons in the hippocampus [87, 88]. The lack of effect of indomethacin on NOR performance could also stem from the 10-day gap between the last drug injection and NOR testing; in other words, it is possible that after the last administration the effects of indomethacin on recognition memory diminish over time and become undetectable at a certain point. Nevertheless, it is important to note that, in the other memory task (IA), where there was a 9-day gap between the last injection and the test session, effects of the drug (and of CSDS) were observed. On the other hand, the high inter-group variabilities (reflected by the SEM bars in Fig 3) could also explain the lack of differences between the NOR discrimination index in the SUS+SAL and SUS+IND groups. Nonetheless, it was confirmed that there were no outliers in our NOR data. Even so, the explanation for the differential effects of the drug must be proposed with caution. In relation to non-stressed mice, we found that indomethacin prevented the formation of emotional memory, whereas it did not disrupt recognition memory. Similarly, it has been reported that indomethacin impaired passive avoidance memory in mice [79] and in chicks [89]. Given that the neurobiological action of indomethacin on specific brain areas involved in these types of memory were not directly evaluated in our study, it is not possible to give a comprehensive explanation for the unexpected differential results on emotional memory in non-stressed and stressed subjects. Therefore, further research is required to better understand the effects of this drug on memory.

In relation to motor activity, stressed mice did not show significant changes in spontaneous locomotor activity. Although several studies have found a decrease in motor activity in defeated animals of several species, such as rats (e.g. [90]), tree shrews (e.g. [91]) and mice (e.g. [92]), our data are in accordance with results obtained previously in our laboratory in mice [19–21]. This lack of motor effects was evident not only in the elevated plus-maze task (in which no differences were observed in the number of closed arms entries), but also in the inhibitory avoidance task, as no statistically significant differences were observed among the groups in terms of their training latencies. Therefore, it can be concluded that the effects of CSDS on memory were not secondary to the motor effects of stress.

In this study, CSDS induced different levels of anxiety in stressed and non-stressed animals, as anxiety-like behavior was heightened in socially defeated mice (i.e., lower percentage of open arm entries). There is increasing evidence of enhancement of anxiety-like behaviors in rodents, as a consequence of social stress, when measured in the elevated plus-maze test [8, 19–21, 37, 51, 93, 94], although the absence of this anxiogenic effect has also been reported [19, 95]. Similar to our results, other studies have found that defeated mice from both the susceptible and unsusceptible groups displayed anxiety-like behavior in the elevated plus-maze test (e.g. [37, 96, 97]) as well as in other anxiety tests, such as the open field test (e.g. [96]) and the light/dark preference test (e.g. [98]). It is possible that the lack of difference in anxiety between resilient and susceptible animals stems from the large within-group variability. Nevertheless, we ran a further analysis distinguishing a third group of “in-between” animals by clusters based on the SI Ratio, and the three stressed groups also showed higher anxiety (without significant differences between them) than the non-stressed mice. Anxiety could act as a confounding factor in the effects of CSDS on memory. On the one hand, the higher level of anxiety/fear observed in stressed animals in this study could have resulted in these subjects taking longer to enter the dangerous compartment; as a consequence, their IA performance would have improved. However, our stressed mice did not display IA learning, despite their enhanced anxiety. On the other hand, this higher level of anxiety in the stressed mice could have resulted in them being more active and having lower test latencies. Nevertheless, it is important to note that there were no significant differences in locomotor activity between stressed and non-stressed mice in either the elevated plus maze or with respect to training latencies of IA. Therefore, we believe that the effects of CSDS on memory are not secondary to its effects on anxiety. In this study, no effects of chronic indomethacin treatment were observed in either stressed or non-stressed animals. Little is known about the effects of indomethacin on anxiety, but Smith et al. [99] reported that indomethacin had no significant effect on the cardiovascular response of conscious lambs to lipopolysaccharide. Therefore, it is not surprising that our study observed the inefficacy of indomethacin in reducing symptoms of anxiety in stressed mice.

Pain research has shown that not all noxious stimuli are processed centrally or peripherally in the same way and not all aversive stimuli are capable of eliciting an analgesic response, but can, in fact, elicit a hyperalgesic response [100]. Indeed, stress can affect pain perception differentially, as accession of hyperalgesia or hypoalgesia depends on the type of stressor, as well as its intensity and duration [101, 102]. It has been argued that this dual action of stress–exacerbation vs. inhibition–on pain modulation depends to a great extent on pre-existing conditions, in particular previous pain experience associated with methodological factors, along with previous adverse life events [103]. In our study, pain sensitivity in chronically defeated mice was indistinguishable from that in non-defeated animals. In the same way, Aghajani et al. [104] reported that pain behavior in an unstable group (whose cage-mate was changed) did not differ from control animals. Therefore, similar to the rationale applied to motor and emotional effects, we believe that the effects of CSDS on emotional memory are not secondary to its effects on pain sensitivity.

As with any research, our study has its limitations and strengths. From our perspective, the main limitation is the lack of stress hormones measures (e.g. corticosterone levels) and other physiological measures, such as inflammation parameters (e.g. prostaglandins levels)–peripherally and centrally–that can be correlated with the behavioral data to determine the specific actions of indomethacin. Establishing the relationships between endocrine system, immune system and behavior will improve our knowledge about the real contribution of the agents mediating the effects of stress. This would obviously allow a more comprehensive interpretation of our results. These measures were not taken due to conditions in our laboratory in terms of facilities and resources necessary for performing such physiological analyses. Nevertheless, we plan to carry out future studies including these measures (in collaboration with other labs where necessary). Another limitation stems from the order of tasks: when a set of behavioral tests is run, the results of each task can be influenced by experience acquired in the previous tests of the battery. Therefore, in our study, the NOR performance could have been biased by the previous IA experience. In fact, the IA test can in itself be stressful, and an inverted U-shaped relationship between footshock intensity and memory performance has been reported [105]. Nevertheless, it is important to note that the footshock intensity used in our study (0.3 mA) was lower than the minimum intensity (0.6 mA) used in the referenced study. Furthermore, the corticosterone levels in the animals receiving the lowest intensity were not significantly higher than those in naïve animals [105]. Based on this rationale, with IA being the main memory test in our line of research, we chose to perform the memory tasks in that order (IA before NOR), acknowledging that other studies have carried out these tasks in the same order (e.g. [106, 107]). In respect of the strengths of this study, we believe it represents a development in the study of pharmacological treatment of the effects of CSDS on memory. Our results show that treatment with the anti-inflammatory indomethacin is effective in attenuating CSDS-induced emotional memory impairment. Furthermore, we have importantly controlled several potential confounding factors (locomotor activity, emotionality and pain sensitivity) through complementary tasks.

## Conclusions

This study shows that: i) Social stress impairs both emotional memory (inhibitory avoidance test) and recognition memory (novel object recognition task) in susceptible subjects; ii) Social stress induces anxiety in post-pubertal mice; iii) The effects of social stress on emotional memory, but not on recognition memory and anxiety, are reversed by indomethacin; iv) The detrimental effects of social stress on emotional and recognition memory are not secondary to the effects of stress on locomotor activity, emotionality or pain sensitivity; v) The social interaction test is confirmed to be a useful tool for distinguishing between subjects that are resilient or susceptible to the effects of CSDS; vi) CD1 is a valid strain of mice to use as stressed subjects in the CSDS protocol, therefore confirming previous evidence.

## Supporting information

S1 Fig Interaction ratios distribution. (TIFF)

S1 Dataset Social interaction data. (XLSX)

S2 Dataset Inhibitory avoidance data. (XLSX)

S3 Dataset Novel object recognition data. (XLSX)

S4 Dataset Elevated plus maze data. (XLSX)

S5 Dataset Hot plate data. (XLSX)
