# Supplementary figures and images for: Indomethacin counteracts the effects of chronic social defeat stress on emotional but not recognition memory in mice
Source: PLoS One. 2017 Mar 9;12(3):e0173182. doi: 10.1371/journal.pone.0173182 (PMC5344348; doi:10.1371/journal.pone.0173182)

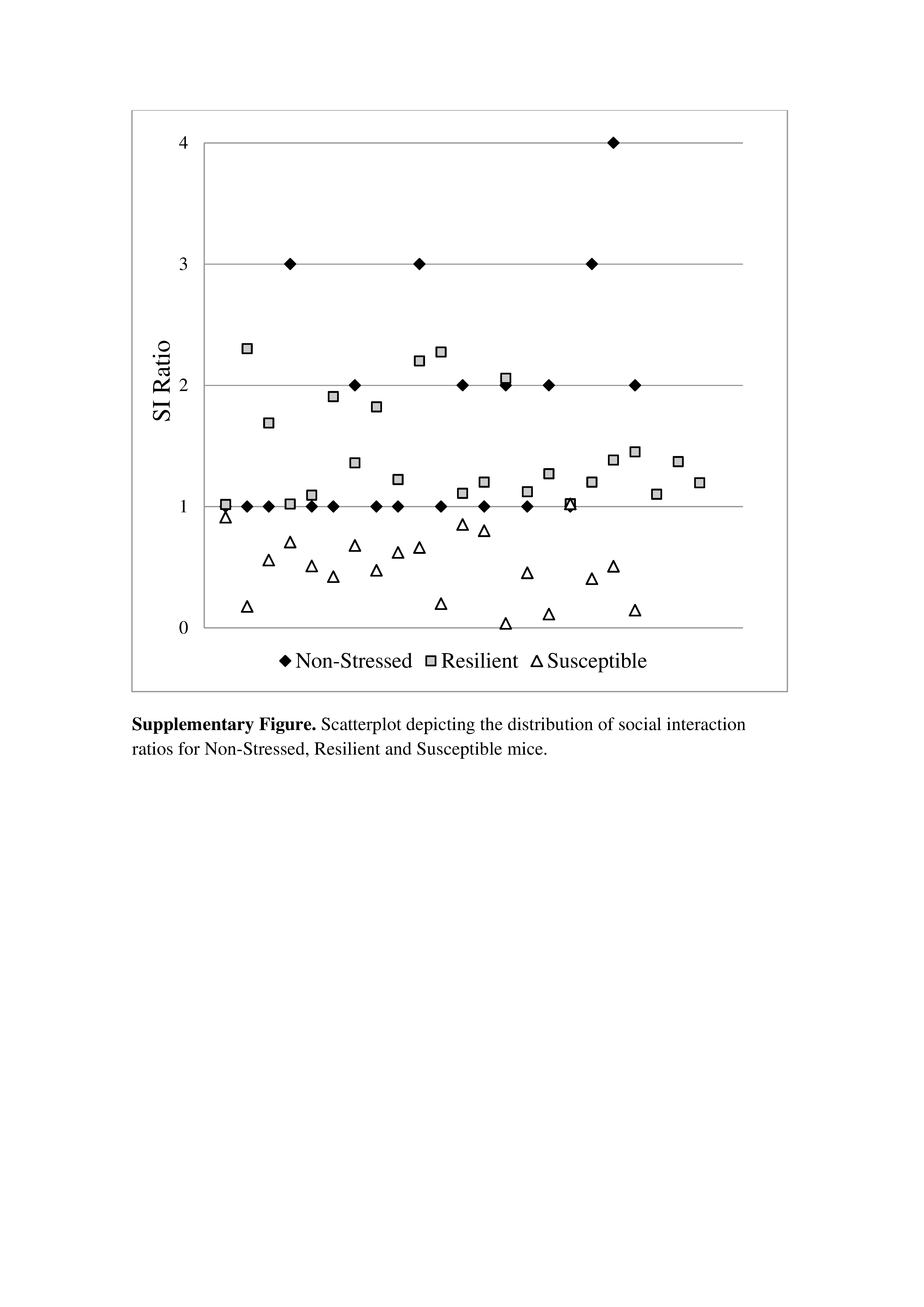

Supplement: S1 Fig — (TIFF) [file pone.0173182.s001.tiff]
